# A consequence of immature breathing induces persistent changes in hippocampal synaptic plasticity and behavior: a role of prooxidant state and NMDA receptor imbalance

**DOI:** 10.3389/fnmol.2023.1192833

**Published:** 2023-06-29

**Authors:** Alejandra Arias-Cavieres, Alfredo J. Garcia

**Affiliations:** ^1^Institute for Integrative Physiology, The University of Chicago, Chicago, IL, United States; ^2^Department of Medicine, Section of Emergency Medicine, The University of Chicago, Chicago, IL, United States; ^3^University of Chicago Neuroscience Institute, University of Chicago, Chicago, IL, United States

**Keywords:** NMDA receptor, intermittent hypoxia (IH), apnea of prematurity, synaptic plasticity, oxidative stress

## Abstract

Underdeveloped breathing results from premature birth and causes intermittent hypoxia during the early neonatal period. Neonatal intermittent hypoxia (nIH) is a condition linked to the increased risk of neurocognitive deficit later in life. However, the mechanistic basis of nIH-induced changes to neurophysiology remains poorly resolved. We investigated the impact of nIH on hippocampal synaptic plasticity and NMDA receptor (NMDAr) expression in neonatal mice. Our findings indicate that nIH induces a prooxidant state that leads to an imbalance in NMDAr subunit composition favoring GluN2B over GluN2A expression and impairs synaptic plasticity. These consequences persist in adulthood and coincide with deficits in spatial memory. Treatment with an antioxidant, manganese (III) tetrakis (1-methyl-4-pyridyl)porphyrin (MnTMPyP), during nIH effectively mitigated both immediate and long-term effects of nIH. However, MnTMPyP treatment post-nIH did not prevent long-lasting changes in either synaptic plasticity or behavior. In addition to demonstrating that the prooxidant state has a central role in nIH-mediated neurophysiological and behavioral deficits, our results also indicate that targeting the prooxidant state during a discrete therapeutic window may provide a potential avenue for mitigating long-term neurophysiological and behavioral outcomes that result from unstable breathing during early postnatal life.

## Highlights

- Untreated immature breathing leads to neonatal intermittent hypoxia (nIH).
- nIH promotes a prooxidant state associated with increased HIF1a activity and NOX upregulation.
- The nIH-dependent prooxidant state leads to NMDAr remodeling of the GluN2 subunit to impair synaptic plasticity.
- Impaired synaptic plasticity and NMDAr remodeling caused by nIH persist beyond the critical period of development.
- A discrete window for antioxidant administration exists to effectively mitigate the neurophysiological and behavioral consequences of nIH.

## Introduction

Human neonates born prematurely (< 37 weeks of gestation) are at an increased risk of apnea of prematurity, a condition that results in breathing instabilities, causing intermittent hypoxemia and, subsequently, oxidative stress (Di Fiore and Vento, 2019; Di Fiore and Raffay, 2021). While instabilities due to immature breathing resolve with continued postnatal development, the occurrence of oxidative stress during early life is hypothesized to be a principal contributor to causing disturbances in the neonatal brain (Panfoli et al., 2018). These disturbances may lead to the emergence of neurobehavioral deficits associated with intellectual disability and autism spectrum disorder in humans (Poets, 2020). Oxidative stress occurs in animal models exposed to perinatal IH (Souvannakitti et al., 2009, 2010; Garcia et al., 2016). In addition, perinatal IH also causes anatomical and neurophysiological changes that are associated with deficits in affective and cognitive behaviors later in life (Cai et al., 2011; Goussakov et al., 2019; Vanderplow et al., 2022). However, the extent to which the prooxidant state contributes to neurophysiological and behavioral changes caused by nIH remains poorly resolved.

The NMDAr has a well-documented role in influencing age-related changes to neurophysiology and behavior. The NMDAr undergoes developmental changes in subunit composition, where GluN2A subunit expression progressively increases and surpasses GluN2B to become the predominant GluN2 isoform in adulthood (Paoletti et al., 2013). As GluN2 subunit composition is an important determinant of both biophysical and downstream signaling properties of the NMDAr (Paoletti and Neyton, 2007; Paoletti et al., 2013; Hansen et al., 2018), disruption to the normal transition of GluN2 subunit composition may affect neurophysiological properties and neurocognition later in life.

In this study, we characterized the immediate and long-term consequences of nIH on the hippocampus and behavioral performance related to spatial learning and memory. We demonstrated that the IH-dependent prooxidant state is linked to dysregulation in GluN2 subunit composition during development, which is associated with impaired NMDAr-dependent synaptic plasticity. These phenomena continue later in life and coincide with deficits related to spatial memory. While administration of the superoxide anion scavenger, MnTMPyP, during nIH prevents these deficits, treatment following nIH was ineffective. These findings indicated that nIH-dependent oxidative stress has a central role in perturbing the normal developmental trajectory of NMDAr-dependent physiology and behavior later in life. Furthermore, we identified a previously undescribed interventional developmental window effective for mitigating the impact of nIH that causes long-lasting consequences on neurophysiology and behavioral changes.

## Materials and methods

### Study approval

All animal protocols were approved by the Institutional Animal Care and Use Committee of The University of Chicago, in accordance with the National Institute of Health guidelines.

### Animals

C57BL/6 mice of both sexes (P4 to P60) were used in this study. They were housed in AAALAC-approved facilities with a 12-h/12-h light-dark cycle and had *ad libitum* access to food and water. To ensure sufficient neonatal nutrition, litters were culled down to six pups prior to the start of experimental protocols. The dam and pups were kept together during all *in vivo* experimental protocols, allowing unrestricted access to nutrition for the offspring.

### *In vivo* experimental protocols

Five *in vivo* experimental protocols were used in this study.

#### Protocol 1

**Figure 1:**
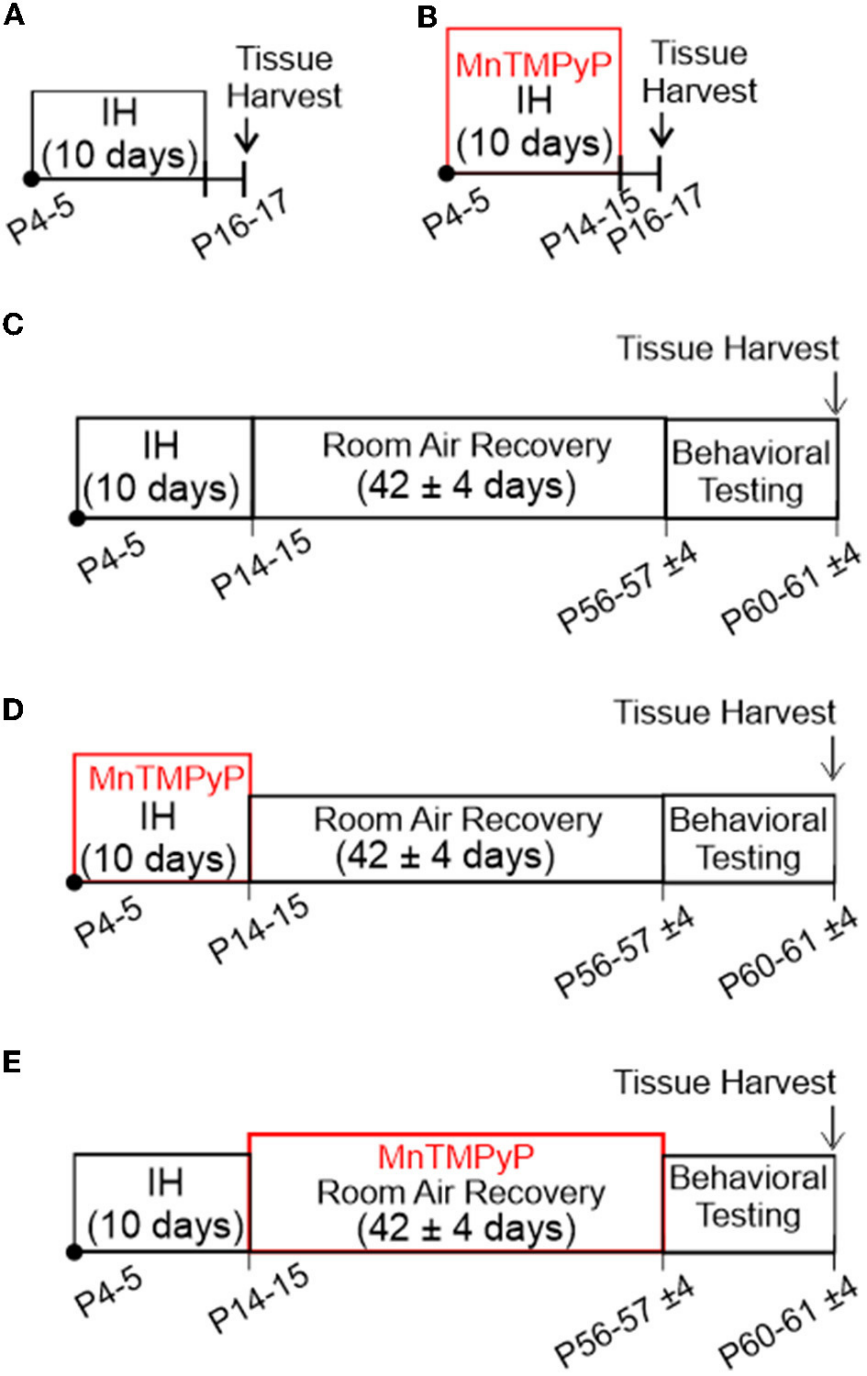
*In vivo* experimental protocols used in this study. **(A)** Exposure to neonatal intermittent hypoxia (nIH). Mice (P4-5) were exposed to intermittent hypoxia (IH) for 10 days. Mice were euthanized for tissue harvest 24 to 48 h following exposure. **(B)** MnTMPyP administration during nIH (nIH_Mn_). Neonatal mice were treated with a single daily dose of MnTMPyP during exposure to nIH [see **(A)**]. **(C)** Adult mice exposed to IH during neonatal life (Adult_nIH_). Neonatal mice were exposed to nIH [see **(A)**] and were then allowed to recover and develop for ~6 weeks in room air. At the end of the recovery period, the mice underwent behavioral testing and were subsequently euthanized for tissue harvest. **(D)** Adult mice exposed to IH and treated with MnTMPyP during neonatal life (Adult_nIH − Mn_). Neonatal mice were exposed to nIH_Mn_ [see **(B)**] and were then allowed to recover and develop for ~6 weeks in room air. At the end of the recovery period, the mice underwent behavioral testing and were subsequently euthanized for tissue harvest. **(E)** Adult mice that underwent MnTMPyP treatment following IH exposure during neonatal life (Adult_REC − Mn_). Neonatal mice were exposed to nIH [see **(A)**] and were treated with daily MnTMPyP during the room air recovery period (Adult_REC − Mn_). Behavioral testing was performed at the end of the recovery period followed by tissue harvest at the end of the recovery period.

Exposure to neonatal intermittent hypoxia (nIH, Figure 1A). Mice (P4-5) were exposed to intermittent hypoxia (IH). IH was achieved as previously described (Peng and Prabhakar, 2003; Garcia et al., 2016). In brief, IH was executed during the light cycle and lasted for 8 ± 1 h per day for ten consecutive days. Exposure to nIH began on P4 to P5. Each hypoxic cycle was achieved by flowing 100% N_2_ into the chamber for approximately 60 s until a hypoxic state (nadir chamber O_2_: 4.5 ± 1.5%) was achieved for approximately 10 s, which was immediately followed by reoxygenation and air break (19 ± 2.5% O_2_; duration 300 s). Following the ten days of IH, mice were euthanized for tissue harvest 24–48 h following exposure. Age-matched mice were reared in room air until tissue harvest, during which they were used as control (control).

#### Protocol 2

MnTMPyP administration during nIH (nIH_Mn_, Figure 1B). Neonatal mice were treated with a single daily dose of MnTMPyP (Enzo Life Sciences, Cat #ALX-430–070) during exposure to nIH (i.e., Protocol 1). Neonatal mice treated with saline instead of MnTMPyP during nIH were used as the control group (nIH_saline_).

#### Protocol 3

Adult mice exposed to IH during neonatal life (Adult_nIH_, Figure 1C). Neonatal mice were exposed to nIH (i.e., Protocol 1) and were then allowed to recover and develop for ~6 weeks (42 ± 4 days) in room air. At the end of the recovery period, the mice underwent behavioral testing and were subsequently euthanized for tissue harvest. Age-matched mice exposed only to room air were used as control (Adult_control_).

#### Protocol 4

Adult mice exposed to IH and treated with MnTMPyP during neonatal life (Adult_nIH − Mn_, Figure 1D). Neonatal mice were exposed to nIH_Mn_ (i.e., Protocol 2) and were then allowed to recover and develop for approximately 6 weeks (42 ± 4 days) in room air. At the end of the recovery period, the mice underwent behavioral testing and were subsequently euthanized for tissue harvest.

#### Protocol 5

Adult mice that underwent MnTMPyP treatment following IH exposure during neonatal life (Adult_REC − Mn_, Figure 1E). Neonatal mice were exposed to nIH (i.e., Protocol 1) and were treated with daily MnTMPyP (42 ± 4 days) during the room air recovery period (Adult_REC − Mn_). Behavioral testing was performed at the end of the recovery period followed by tissue harvest at the end of the recovery period.

All mice treated with MnTMPyP received a single daily dose (5 mg • kg^−1^ *i.p*.) at the beginning of each day for the treatment regimens. MnTMPyP dosing was based on a previous study (Arias-Cavieres et al., 2020, 2021; Khuu et al., 2021). Body mass immediately following IH and 6 weeks following IH exposure were similar to age-matched controls (Table 1).

**Table 1:** Control and nIH body mass at the postnatal age.

**Postnatal day**	**Control (*N*)**	**nIH and nIH_Saline_ (*N*)**	**nIH_Mn_ (*N*)**	**nIH_REC − Mn_ (*N*)**	***P*-value**
4–5	2.59 ± 0.06 (42)	2.48 ± 0.08 (38)	2.43 ± 0.08 (36)	2.52 ± 0.10 (13)	*P* = 0.50
14–15	6.39 ± 0.12 (42)	6.58 ± 0.27 (38)	6.46 ± 0.20 (36)	6.18 ± 0.13 (13)	*P* = 0.57
55–60	28.32 ± 0.78 (14)	29.49 ± 0.66 (20)	28.78 ± 0.60 (16)	39.98 ± 0.87 (13)	*P* = 0.92

### Hippocampal brain slice preparation

Coronal hippocampal slices were prepared from mice either 24 h following the end of nIH (P14 to P15) or ~6 weeks after nIH (P55 to P60). Mice were anesthetized with isoflurane and euthanized by rapid decapitation. The brain was rapidly harvested and blocked, rinsed with cold artificial cerebrospinal fluid (aCSF), and mounted for vibratome sectioning. The mounted brain tissue was submerged in aCSF (4°C; equilibrated with 95% O_2_, 5% CO_2_), and coronal cortico-hippocampal brain slices (350 μm thick) were prepared. Slices were immediately transferred into a holding chamber containing aCSF equilibrated with 95% O_2_ and 5% CO_2_ (at 20.5 ± 1°C). Slices were allowed to recover a minimum of 1 h prior to the transfer into a recording chamber. Slices were used within 8 h following tissue harvest, with an aCSF composition (in) of 118 mM NaCl, 10 mM glucose, 20 mM sucrose, 25 mM NaHCO_3_, 3.0 mM KCl, 1.5 mM CaCl_2_, 1.0 mM NaH_2_PO_4_ and 1.0 mM MgCl_2_. The osmolarity of aCSF was 305–315 mOsm/L, and when equilibrated with 95% O_2_/5% CO_2_, the pH was 7.42 ± 0.2.

### Extracellular recording of the field excitatory postsynaptic potential

The extracellular recording of the fEPSP was established in aCSF (31.0 ± 2°C, equilibrated with 95% O_2_ 5% CO_2_), which was superfused and recirculated over the preparation. A custom-constructed bipolar stimulation electrode composed of twisted Teflon-coated platinum wires (wire diameter: 127 μm, catalog number 778000, AM Systems, Washington, WA) was used to electrically evoke the fEPSP. The stimulation electrode was positioned in the Schaffer collaterals, and a recording electrode (< 2 MΩ) was positioned into the *stratum radiatum* of area CA1. The intensity of the electrical current (100–400 μA; 0.1–0.2 ms duration) was set to the minimum intensity required to generate 50% of the maximal fEPSP.

The fEPSP was evoked every 20 s. After 10 min of baseline recording, LTP was induced using Theta burst stimulation (TBS), which consisted of four trains of 10 bursts at 5 Hz, each burst was comprised of four pulses at 100 Hz. Recordings continued for up to 1 h. The fEPSP slope was normalized to baseline values. 50 μM D-AP5 or 100 μM D-L AP5 (Sigma-Aldrich, Saint Louis, MO; Cat#A5282) was used to block NMDAr, TCN-213 (5 μM, Tocris Bioscience, Bristol, UK; Cat#4163) was used to block GluN2A, and ifenprodil (5 μM, Tocris Bioscience, Bristol, UK; Cat#0545) was used to block GluN2B. The current stimulus was set at the minimum current value (150 to 250 μA) required to evoke the initial fEPSP at a maximal value of 50%. Recordings were made using either a MultiClamp 700B (Molecular Devices, San Jose, CA, USA) or using a differential amplifier (AM system, Washington, DC, USA).

### Nuclear western immunoblot assay

Samples for immunoblot assays were prepared from the isolated hippocampus that was homogenized using N-PER (Thermo Fisher Scientific, Cat#87792; Whaltam, MA) in ice following manufacturer instructions. Briefly, tissues were homogenized in the cytoplasmic extraction buffer. After the isolation of the cytoplasmic fragment, the insoluble pellet containing nuclear proteins was suspended in a nuclear extraction buffer and separated by centrifugation (16,000 rcf). Halt™ Protease Inhibitor Cocktail (Thermo Fisher, Whaltam, MA; Cat#78429) was added into cytoplasmic and nuclear extraction buffers to prevent protein degradation. Samples were boiled for 15 min in a loading buffer (Bio-Rad, Hercules, CA, USA), denaturated by β-mercaptoethanol at 75°C before loading 50–60 μg protein onto 4–20% Mini-PROTEAN TGX Stain-FreeTM Protein Gels (Bio-Rad, Hercules, CA, USA), and electrophoresed at 120 V for 120 min. Then, gels were transferred to PVDF membrane (Bio-Rad) using the Transfer-Blot Turbo System (Bio-Rad, Hercules, CA). Membranes were subsequently blocked for 2 h at room temperature with 5% bovine serum albumin (BSA) (Sigma-Aldrich, MN, USA) in Tris-buffered saline (TBS) (Bio-Rad, Hercules, CA, USA). Membranes were then incubated under constant shaking with primary antibodies for HIF1a (1:500; Abcam, Bristol, UK; Cat# ab1, RRID:AB_296474) and TBP (1:2000; Danvers, MA; Cat# 44059, RRID:AB_2799258). After washing three times with 0.3% TBS-Tween for 20 min, the membranes were incubated for 1.5 h at room temperature with appropriate secondary antibodies. Finally, the membranes were washed three times with 0.3% TBS-Tween for 20 min, and immunoreactive proteins were detected with Super Signal^TM^ West Femto reagents according to the manufacturer's instructions (Thermo Fisher Scientific, Whaltam, MA; Cat#34095). Signals were captured with the ChemiDoc Imaging system (Bio-Rad, Hercules, CA). The ImageJ program (National Institutes of Health, USA) was used to quantify optical band intensity.

### Whole-cell Western immunoblot assay

Samples for immunoblot assays were prepared from the isolated hippocampus that was homogenized using M-PER^TM^ (Thermo Fisher Scientific, Whaltam, MA; Cat#78501) and Halt Protease Inhibitor Cocktail (Thermo Fisher Scientific, Whaltam, MA; Cat#78429) kept on ice. Samples were centrifuged at 12,000 rpm for 15 min at 4°C, and the pellet was discarded. Samples were boiled for 15 min in a loading buffer (Bio-Rad, Hercules, CA), denaturated by β-mercaptoethanol at 75°C before loading 20–30 μg protein onto 4–20% Mini-PROTEAN TGX Stain-FreeTM Protein Gels (Bio-Rad, Hercules, CA), and electrophoresed at 120 V for 120 min. Then, gels were transferred to PVDF membrane (Bio-Rad) using the Transfer-Blot Turbo System (Bio-Rad, Hercules, CA). Later, membranes were subsequently blocked for 2 h at room temperature with 5% non-fat milk (Bio-Rad, Hercules, CA) or 5% bovine serum albumin (BSA) (Sigma-Aldrich, MN, USA) in Tris-buffered saline (TBS) (Bio-Rad, Hercules, CA). Membranes were incubated under constant shaking with primary antibodies for GluN1 (1:2000; Abcam, Bristol, UK; Cat# ab109182, RRID:AB_10862307), GluN2A (1:2000; Danvers, MA; Cat# 4205, RRID:AB_2112295), GluN2B (1:2000; Danvers, MA; Cat# 14544, RRID:AB_2798506), NOX2 (1:2000; Abcam, Bristol, UK; Cat# ab129068, RRID:AB_11144496), NOX4 (1:500; Saint Louis, MO; Cat# NB110-58841B, RRID:AB_1217375), and GAPDH (1:10000; Abcam, Bristol, UK; Cat# ab8245, RRID:AB_2107448). Incubations were performed at 4°C overnight in 5% non-fat milk or BSA. After washing three times with 0.2% TBS-Tween for 15 min, the membranes were incubated for 1.5 h at room temperature with appropriate secondary antibodies. Finally, the membranes were washed three times with TBS-Tween 0.2% for 15 min and immunoreactive proteins were detected with enhanced chemiluminescence reagents according to the manufacturer's instructions (Bio-Rad, Hercules, CA). Signals were captured with the ChemiDoc system (Bio-Rad, Hercules, CA). The ImageJ program (National Institutes of Health, USA) was used to quantify optical band intensity.

### TBARS assay

Whole-cell protein lysates were isolated from the entire hippocampal tissues using the RIPA buffer (Thermo Fisher Scientific, Catalog No. R0278) in the presence of protease and phosphatase inhibitors (Thermo Fisher Scientific, Catalog No. 78429) kept on ice. Protein lysates were immediately processed and stored at −80°C until use. The amount of lipid-peroxidation was determined using a TBARS Assay Kit (Cayman Chemical, Cat#10009055) as per manufacturer's instructions. Absorbance was measured between 530 and 540 nm using a plate reader. The analysis for determining MDA values was conducted in accordance with the manufacturer's instructions.

### Barnes maze

The Barnes maze test was performed using a custom-made opaque white circular acrylic platform (92.4 cm in diameter) with 20 equidistant holes (5.08 cm in diameter and 2.54 cm from the edge). The platform was elevated (30 cm from the floor) from ground and surrounded by four identical walls (27.94 cm high). By default, each hole was closed with a fixed piece of opaque acrylic that could be removed to lead to a dark exit box. Lighting was achieved through diffuse overhead fluorescent lighting such that all holes were equally lit. An overhead camera was suspended above the maze. Data collection and *post-hoc* analysis were performed using CinePlex Digital Video Recording/Tracking System (Plexon, Dallas, TX).

As described in a previous study (Arias-Cavieres et al., 2020), the task was performed using a 4-day protocol consisting of one training trial per day for 3 consecutive days and a PROBE trial on the fourth day. The Barnes maze protocol began after 6-week exposure to IH with respective controls run at the same time. For the training trials, all but one of the holes (i.e., the exit zone) were closed. An exit box with a small ramp was placed directly underneath the exit zone. Animals were given a maximum of 6 min to locate the exit, and if unable to locate the exit, they were gently guided to the exit. If the mouse found and entered the exit before the given duration of 6 min were over, the trial ended at the time that the mouse entered the exit and the mouse was promptly returned to its home cage. During the PROBE trial, all holes were closed, and the animal was given 6 min to explore the maze. To reduce potential confounders due to odor, the arena was sanitized with 75% ethanol between trials.

Entry probability for each hole during the PROBE trail was calculated by the following:


$$\begin{array}{l} EP_{n}\;=100\%\;\times \;\frac{X_{n}}{X_{total}} \end{array}$$

where *EP*_*n*_is the entry probability for hole n; *x*_*n*_is the number of entries into hole n; and *x*_*total*_ is the sum of entries across all holes during the PROBE trial.

### Object location task

The Object Location task was performed using a modified protocol described by Wimmer et al. (2012). The procedure included three phases: open field, familiarization, and object location. Each phase was performed in the acrylic open arena (W: 33 cm, L: 36 cm, H: 33 cm). During the open field phase, the mouse was habituated and allowed to freely explore the arena for 10 min. The familiarization session consisted of three consecutive trials (session duration: 5 min; intersession duration: 5 min), where the mouse was placed in the arena and allowed to explore two different objects (i.e., object A and object B). The time for exploring both objects was recorded. The object location phase occurred 24 h following familiarization, where one object presented in the familiarization session was repositioned (B). The time taken for exploring the repositioned object (object B) was compared between experimental groups. Exploration time was expressed as a percentage describing the proportion of time the subject explored object B in relation to total exploration time.

To eliminate odor cues between trials, the experimental apparatus and all objects were cleaned with 75% ethanol after each trial. The behavior of mice was recorded with a video camera positioned over the behavioral apparatus, and the collected videos were analyzed with the ANY-MAZE software (Stoelting Co., Wood Dale, IL, USA).

### Statistical analysis

Statistical analysis was performed using Prism 6 (GraphPad Software, Inc.; RRID:SCR_015807), and the results were plotted in either Prism 6 or Origin 2018b (OriginLab, RRID:SCR_014212). Comparisons between the two groups were conducted using unpaired two-tailed *t*-test with Welch's correction or paired comparisons, where appropriate. The equality of variances between the two groups was determined with an F test. Analyses that involved comparisons beyond more than two groups were assessed using a one-way ANOVA of means followed by a *post-hoc* Bonferroni's multi-comparison test. Data are presented as mean ± S.E.M, and where appropriate, individual responses are overlaid over box plots. The upper and lower limits of each box represented the 25th and 75th percentile of the cohort, respectively, while the inter-limit line within the box represented as median. The box plot error bars represented the maximum and minimum values in the data set. Significance was defined as a *P*-value of < 0.05.

## Results

### nIH promotes a prooxidant state, increases nuclear HIF1a and upregulates NADPH oxidase in the neonatal hippocampus

**Figure 2:**
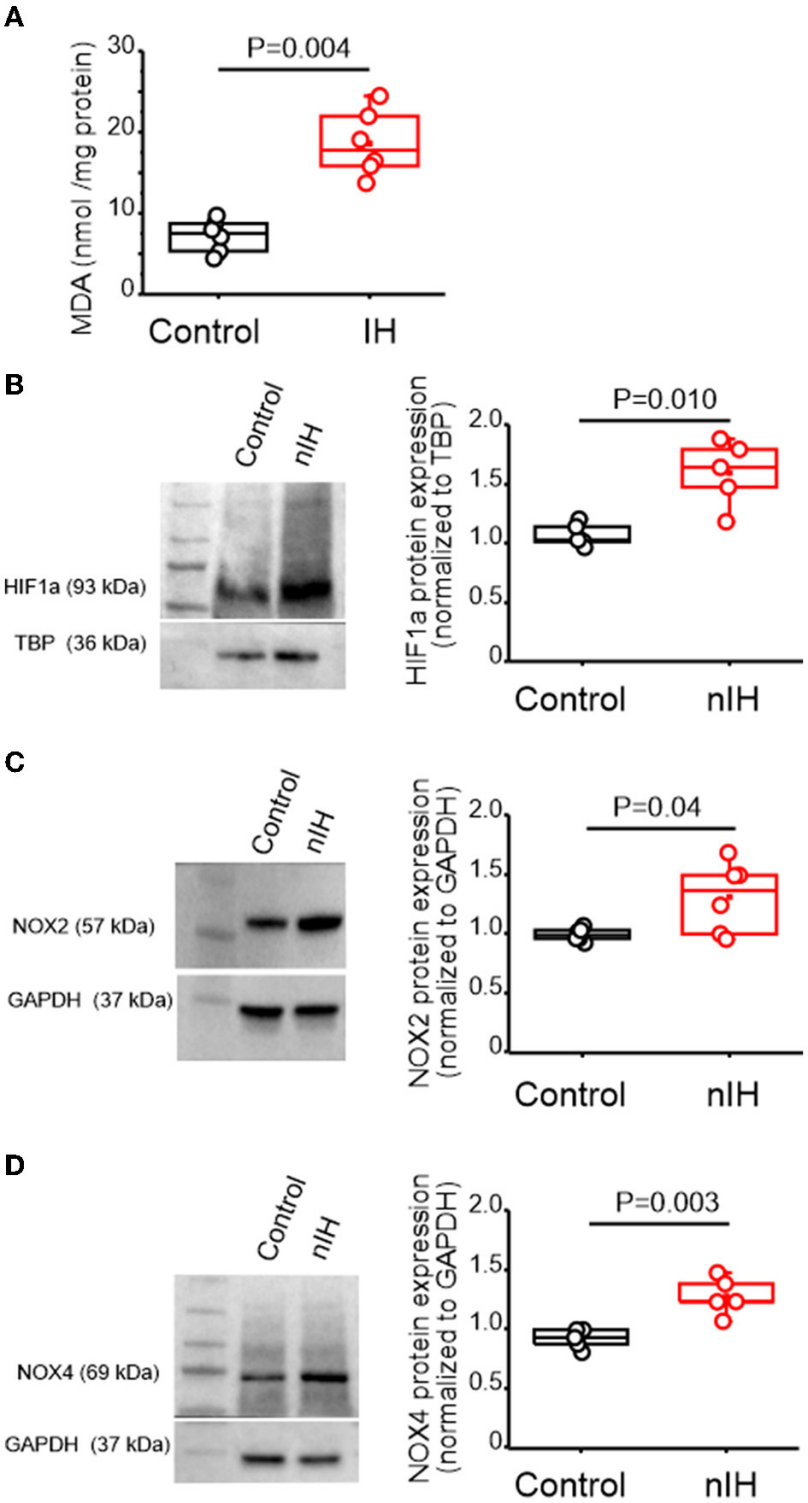
nIH promotes a prooxidant state that coincides with increased nuclear HIF1a and NOX4 isoforms in the neonatal hippocampus. **(A)** Malondialdehyde (MDA) content was measured in hippocampal homogenates from control and nIH (two-tailed t-test, *t* = 6.16; df = 7.33; *P* = 0.004). **(B)** Representative immunoblot and corresponding quantification of nuclear HIF1a from control and nIH (two-tailed *t*-test, *t* = 3.98; df = 4.98; *P* = 0.010). **(C)** Representative immunoblot and corresponding quantification of NOX2 from nIH and control (two-tailed *t*-test, *t* = 2.59; df = 5.3; *P* = 0.04). **(D)** Representative immunoblot and corresponding quantification of NOX4 from control and mice exposed to nIH (two-tailed *t*-test, *t* = 4.53; df = 6.01; *P* = 0.003)_._

Measurements of the malondialdehyde (MDA) content in neonatal hippocampal homogenates revealed that the MDA levels were increased with nIH (Figure 2A, control: 7.2 ± 0.82 nmol · mg^−1^ protein and nIH: 18.59 ± 1.65 nmol · mg^−1^ protein, *P* = 0.004; *N* = 6 preparations per condition). This was accompanied by greater nuclear content of the prooxidant transcription factor, HIF1a (Figure 2B, control = 1.06 ± 0.04 and nIH = 1.59 ± 0.12, *P* = 0.010; *N* = 5 per condition) and increased expression of two isoforms of the prooxidant enzyme NADPH oxidase (NOX), NOX-2 (Figure 2C control = 1.02 ± 0.02 and nIH = 1.31 ± 0.12; *P* = 0.04; *N* = 6 per condition), and NOX-4 (Figure 2D, NOX-4: control = 0.96 ± 0.03 and nIH = 1.30 ± 0.07; *P* = 0.003; *N* = 5 per condition).

### Attenuated synaptic plasticity by nIH is associated with changed NMDAr subunit composition

**Figure 3:**
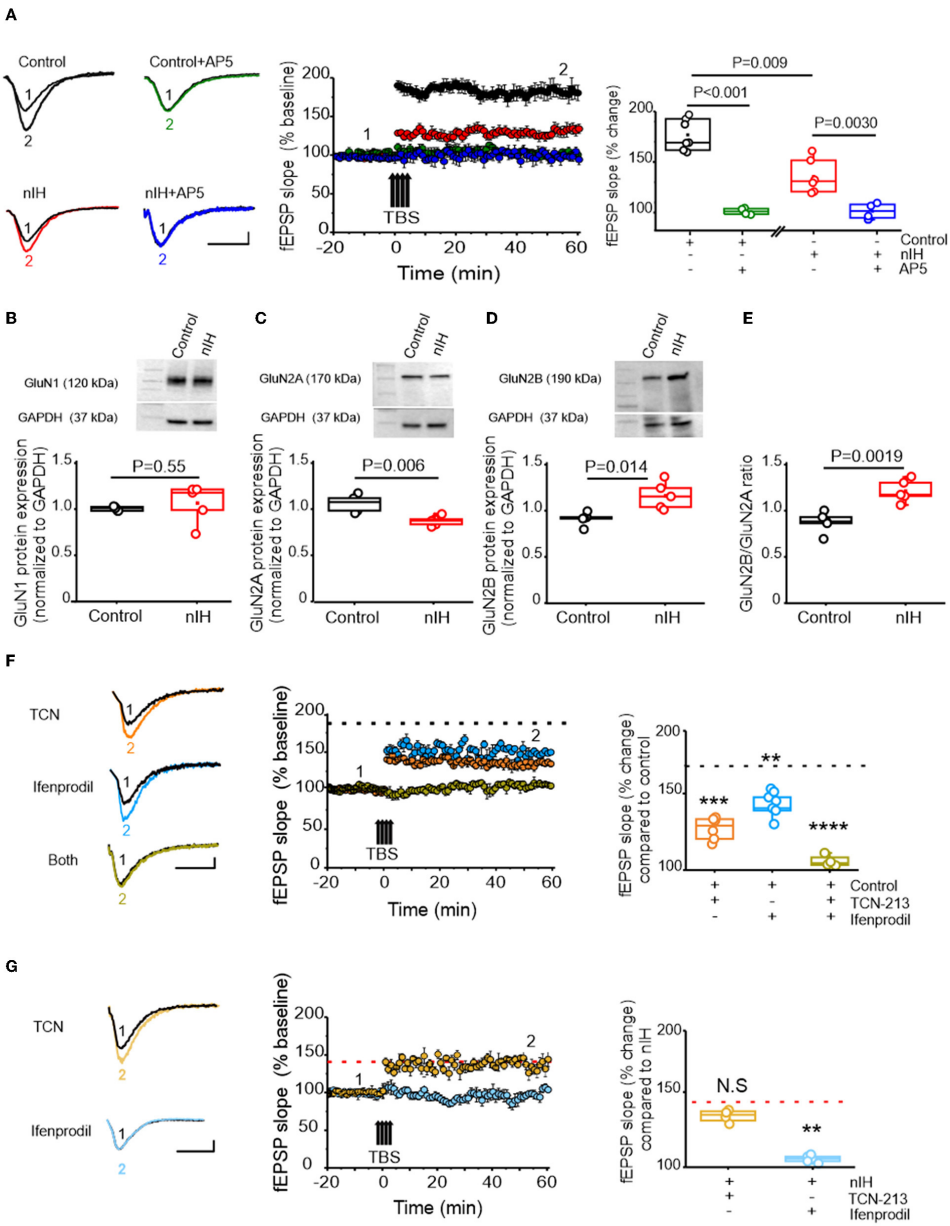
nIH suppresses LTP and changes the sensitivity of LTP to TCN-213 and ifenprodil. **(A)** (left) Representative traces of the evoked fEPSP from control (black), control + AP5 (green), nIH (red), and nIH + AP5 (blue) in baseline conditions prior to (1) and following TBS (2). fEPSP slope plotted as a function of elapsed time relative TBS in control, control + AP5, nIH, and nIH + AP5 and the corresponding comparison of fEPSP slope at 60 min after TBS. **(B)** Representative immunoblot and corresponding comparison of GluN1 in neonatal hippocampal homogenates from control and nIH (two-tailed *t-*test, *t* = 0.63; df = 4.1; *P* = 0.55). **(C)** Representative immunoblot and corresponding comparison of GluN2A in neonatal hippocampal homogenates from control and nIH (two-tailed *t*-test, *t* = 4.017; df = 6.43; *P* = 0.006). **(D)** Representative immunoblot and corresponding comparison of GluN2B in neonatal hippocampal homogenates from control and nIH (two-tailed *t*-test, *t* = 3.43; df = 5.78; *P* = 0.014). (E) Comparison of the GluN2B to GluN2A expression ratios from control and nIH (two-tailed *t*-test, *t* = 4.53; df = 7.97; *P* = 0.0019). **(F)** Representative traces of the evoked fEPSP control +TCN [5 μM] (orange), control + ifenprodil [5 μM] (cyan)], and control + both drugs (olive) in baseline conditions prior to (1) and following TBS (2). fEPSP slope plotted as a function of elapsed time relative TBS for each group and the corresponding comparison of 60 min after TBS (one-way ANOVA, *F* (3.20) = 43.52; *P* < 0.0001). The dashed black line represents the slope of the fEPSP 60 min following TBS in control slices from Figure 2A. **(G)** Representative traces of the evoked fEPSP from nIH + TCN [5 μM] (dark yellow) and nIH + ifenprodil [5 μM] (light blue) in baseline conditions prior to (1) and following TBS (2). fEPSP slope plotted as a function of elapsed time relative TBS for each group and the corresponding comparison at 60 min after TBS [one-way ANOVA, *F* (2.12) = 11.52; *P* = 0.0016]. The red dashed line represents the mean slope of the fEPSP 60 min following TBS in nIH slices from Figure 2A. Scale bars: 10 ms x 0.2 mV. The Bonferroni *post-hoc* test was performed following a one-way ANOVA. **P < 0.01, ***P < 0.001 and ****P < 0.0001.

In adult rodents, IH dependency increased nuclear HIF1a, and a shift toward a prooxidant state is associated with impaired NMDAr-dependent synaptic plasticity (Arias-Cavieres et al., 2020, 2021). Therefore, we next characterized how nIH impacted LTP, evoked by theta-burst stimulation (LTP_TBS_) in neonatal hippocampal slices from control and nIH mice. LTP_TBS_ was evoked in area CA1 in both control (Figure 3A, black, *n* = 7 slices; *N* = 6 mice) and nIH (Figure 3A, red, *n* = 6; *N* = 5) mice, and blockade of NMDAr, using AP5, prevented LTP_TBS_ in both groups (Figure 3A, control (green): 99.74 ± 2.88, *n* = 4, *N* = 4; nIH (blue): 99.07 ± 3.38, *n* = 4, *N* = 4). However, the magnitude of LTP_TBS_ was smaller in nIH slices as compared to control mice (Figure 3A, control: 177.02 ± 5.9% vs. nIH: 135.10 ± 6.8% over baseline, *P* = 0.009). Such a difference may have resulted from an overall downregulation in NMDAr expression or a change in receptor subunit composition.

While the expression of GluN1, the obligatory subunit of the NMDAr, was similar between control and nIH (Figure 3B, control: 1.06 ± 0.01, *N* = 5; nIH: 1.11 ± 0.09, *N* = 5; *P* = 0.55), GluN2 subunit composition was different between the groups. GluN2A expression was reduced following nIH (Figure 3C, control: 1.07 ± 0.04, *N* = 5; nIH: 0.88 ± 0.02, *N* = 5; *P* = 0.006), whereas GluN2B expression following nIH was greater than control (Figure 3D, control: 1.06 ± 0.03; *N* = 5; nIH: 1.38 ± 0.09; *N* = 5, *P* = 0.014). These differences coincided with a larger GluN2B:GluN2A ratio following IH (Figure 3E, *P* = 0.0019). Since GluN2 subunit composition is a significant factor in dictating NMDAr-dependent physiology (Kumar and Huguenard, 2003; Paoletti et al., 2013; Kumar et al., 2019), we sought to determine whether changed LTP_TBS_ following nIH was related to changes in GluN2 subunit composition. In control slices, the magnitude of LTP_TBS_ was reduced when blocking either the GluN2A containing NMDAr with TCN-213 (Figure 3F, orange, 129.87 ± 3.07% over baseline; *n* = 7 slices, *N* = 6) or the GluN2B containing NMDAr with ifenprodil (Figure 3F, cyan, 146.67 ± 3. 29% over baseline; *n* = 6 slices, *N* = 6). When both agents were applied, LTP_TBS_ was not effectively evoked (Figure 3F, olive, 96.37 ± 3.36% over baseline; *n* = 4 slices, *N* = 4; *P* < 0.0001).

Following nIH, LTP_TBS_ appeared to be unaffected by TCN-213 (Figure 3G; light yellow, 137.58 ± 2.24% over baseline; *n* = 4 slices, *N* = 4 mice) when compared to treated nIH slices (from Figure 3A); however, ifenprodil appeared to prevent LTP_TBS_ following nIH (Figure 3G; light blue, 106.13 ± 2.16% over baseline, *n* = 5 slices, *N* = 5; *P* = 0.0016). Collectively, these results indicated that NMDAr-dependent plasticity following nIH is exclusively driven by GluN2B-containing receptors, which is consistent with the nIH-mediated remodeling of GluN2 subunit composition to favor GluN2B expression over GluN2A.

### MnTMPyP administration during IH mitigates molecular, biochemical, and neurophysiological changes in the neonatal hippocampus

**Figure 4:**
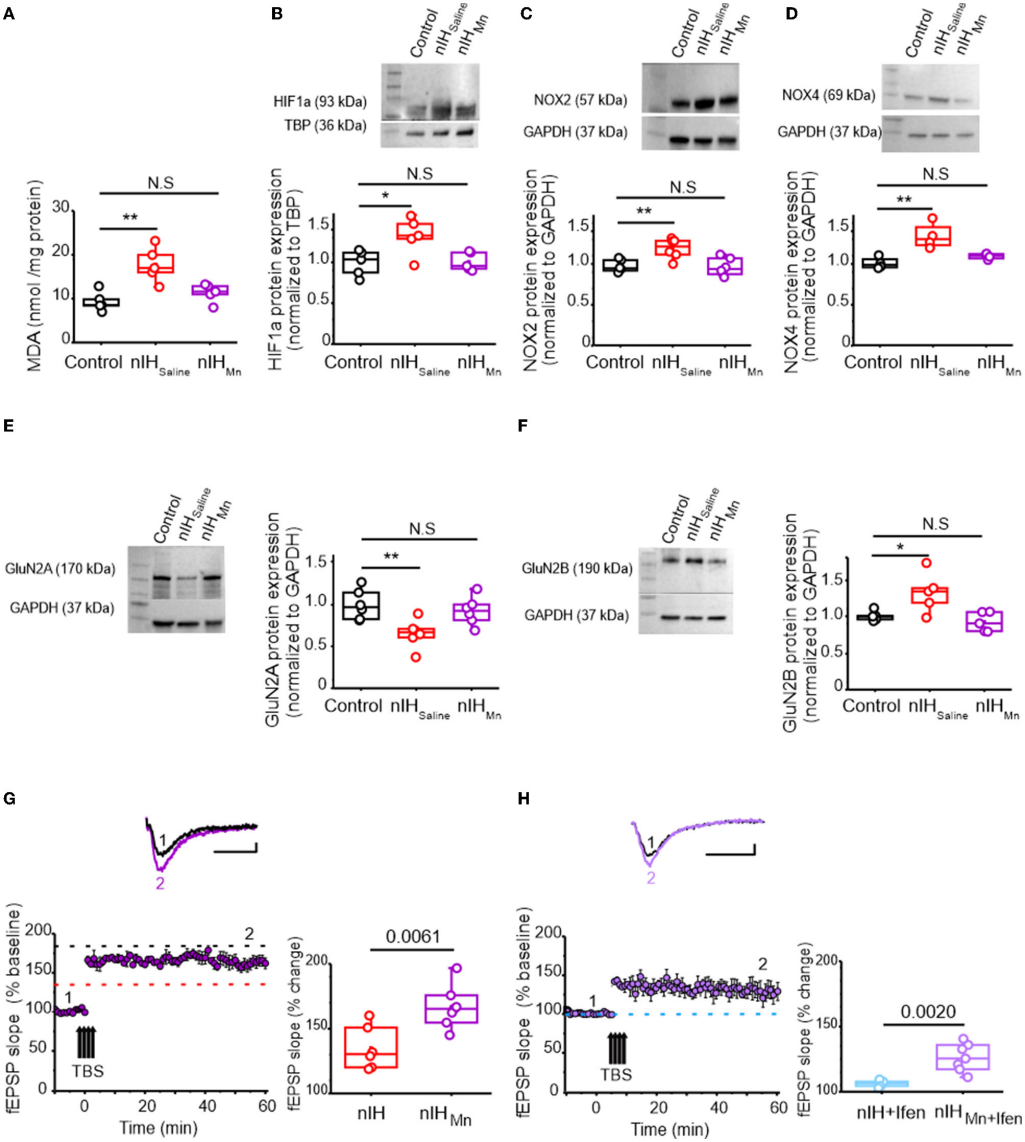
MnTMPyP administration mitigates nIH-mediated oxidative stress, increased nuclear HIF1a, NOX isoform upregulation, and impairments to LTP. **(A)** Comparison of malondialdehyde (MDA) content measured in neonatal hippocampal homogenates from control, nIH_Saline_, and 10-Mn. [one-way ANOVA, *F* (2.12) = 11.53; *P* = 0.0016]. **(B)** Representative immunoblot and corresponding comparison of nuclear HIF1a in neonatal hippocampal homogenates from control, nIH_Saline_, and IH_10 − Mn_ [one-way ANOVA, *F* (2.12) = 5.78; *P* = 0.017]. **(C)** Representative immunoblot and corresponding comparison of nuclear NOX2 in neonatal hippocampal homogenates from control, nIH_Saline_, and IH_10 − Mn_ [one-way ANOVA, *F* (2.15) = 9.26; *P* = 0.0024]. **(D)** Representative immunoblot and corresponding comparison of NOX4 in hippocampal homogenates from control, nIH_Saline_, and nIH_Mn_. [one-way ANOVA, *F* (2.9) = 11.46; *P* = 0.0034]. **(E)** Representative immunoblot and corresponding comparison of GluN2A in homogenates from control, nIH_Saline_, and nIH_Mn_ [one-way ANOVA, *F* (2.15) = 6.63; *P* = 0.0086]. **(F)** Representative immunoblot and corresponding comparison of GluN2B. in homogenates from control, nIH_Saline_, and nIH_Mn_ [one-way ANOVA, *F* (2.12) = 6.88; *P* = 0.01]. **(G)** Representative traces of the evoked fEPSP from nIH_Mn_ (purple) prior to (1) and following TBS (2). fEPSP slope plotted as a function of elapsed time relative to TBS in nIH_Mn_ and the corresponding comparison of fEPSP slope 60 min after TBS in nIH and nIH_Mn_ [two-tailed *t*-test, *t* = 3.41; df = 10.61; *P* = 0.0061]. Dashed lines represent the mean slope of the fEPSP 60 min following TBS in control (black) and nIH (red) slices from Figure 2. **(H)** Representative traces of the evoked fEPSP from nIH_Mn_ in ifenprodil [5 μM] (nIH_Mn+ifen_, light purple) prior to (1) and following TBS (2). fEPSP slope plotted as a function of time elapsed relative to TBS in nIH_Mn+ifen_ and the corresponding comparison of fEPSP slope 60 min after TBS (two-tailed *t*-test, *t* = 4.81; df = 6.97; *P* = 0.0020). The blue dashed line represents the mean slope of the fEPSP 60 min following TBS in ifenprodil-treated nIH slices from Figure 2. Scale bars: 10 ms x 0.2 mV. The Bonferroni *post-hoc* analyses were performed following one-way ANOVA. *P < 0.05, **P < 0.01.

To determine the contribution of the nIH-dependent prooxidant state to the observed changes in the neonatal hippocampus, we administered the superoxide anion scavenger MnTMPyP to a cohort of mice during nIH. While the MDA content in neonatal hippocampi from the control and the cohort receiving MnTMPyP during nIH (nIH_Mn_) were similar, the MDA content in the nIH cohort treated with a saline vehicle (nIH_saline_) was greater than control (Figure 4A; control: 9.31 ± 0.98 nmol per mg protein; nIH_saline_: 17.75 ± 1.78 nmol per mg protein; nIH_Mn_: 11.33 ± 0.93 nmol per mg protein; *P* = 0.0011; *n* = 5 per group). Nuclear HIF1a content (Figure 4B, control: 0.99 ± 0.07; nIH_saline_: 1.32 ± 0.10 and nIH_Mn_: 1.03 ± 0.05, *P* = 0.017; *n* = 5 per group), NOX2 (Figure 4C, control: 1.03 ± 0.03, nIH_saline_: 1.32 ± 0.051 and nIH_Mn_: 0.99 ± 0.046, *P* = 0.0024; *n* = 6 per group), and NOX4 were increased in nIH_saline_ (Figure 4D, control: 1.02 ± 0.03; nIH_saline_: 1.35 ± 0.08 and nIH_Mn_: 1.10 ± 0.01, *P* = 0.0034; *n* = 4 per group) and remaned unchanged in nIH_Mn_. GluN2A expression was also reduced in nIH_saline_ while the expression was unchanged in nIH_Mn_ (Figure 4E, control: 1.08 ± 0.07; nIH_Saline_: 0.68 ± 0.06; nIH_Mn_: 0.92 ± 0.06; *P* = 0.0086; *N* = 6 per group). Furthermore, while GluN2B expression was increased in nIH_saline_, GluN2B expression remained unchanged in nIH_Mn_ (Figure 4F, control: 1.03 ± 0.030; nIH_saline_: 1.38 ± 0.12; nIH_Mn_: 0.92 ± 0.059, *P* = 0.010; *n* = 5 per group). Similarly, the magnitude of NMDAr-dependent LTP in nIH_Mn_ was greater than nIH (Figure 4G, nIH_Mn_: 166.65 ± 6.20% over the baseline; *n* = 7 slices, *N* = 5 mice), and the sensitivity of LTP to ifenprodil was evident when compared to nIH (Figure 4H, 133.26 ± 5.03% over the baseline; *n* = 7, *N* = 5).

### Persistent deficits in NMDAr-dependent synaptic plasticity and GluN2 subunit remodeling is mitigated by MnTMyPyP administration during nIH

**Figure 5:**
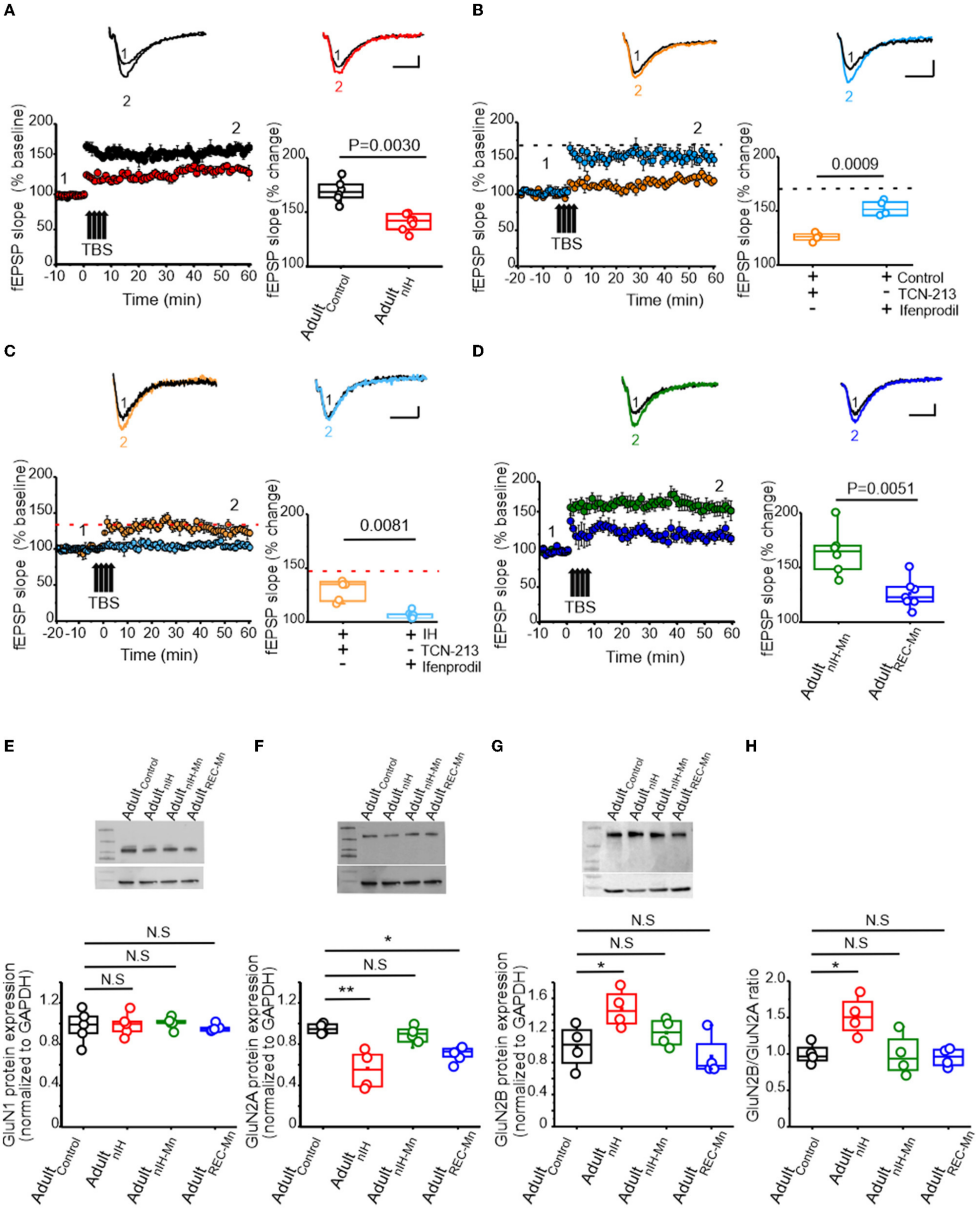
Changes in synaptic plasticity and NMDAr subunit remodeling are present in the adult hippocampus from mice exposed to nIH and can be mitigated by MnTMPyP administration during nIH. **(A)** Representative traces the evoked fEPSP from Adult_control_ (black) and Adult_nIH_ (red) prior to TBS (1) and following TBS (2). fEPSP slope plotted as a function of elapsed time relative to TBS and the corresponding comparison of the fEPSP slope at 60 min following TBS (two-tailed *t*-test, *t* = 5.70; df = 9.04; *P* = 0.003). **(B)** Representative traces of the evoked from control + TCN-213 [5 μM] and control + ifenprodil [5 μM] in baseline conditions prior to (1) and following TBS (2). fEPSP slope plotted as a function of time relative to TBS and the corresponding comparison at 60 min following TBS (two-tailed *t*-test, *t* = 7.43; df = 4.68; *P* = 0.0009). The dashed black dashed line represents the mean fEPSP slope 60 min following TBS in control slices from Figure 4. **(C)** Representative fEPSP trace from Adult_nIH_ + TCN-213 [5 μM] and Adult + ifenprodil [5 μM] prior to (1) and following TBS (2). fEPSP slope plotted as a function of elapsed time relative to TBS and the corresponding comparison at 60 min following TBS (two-tailed *t*-test, *t* = 4.37; df = 4.73; *P* = 0.0081). The dashed red line represents the mean slope in nIH slices from Figure 4A. **(D)** (*top)* Representative traces of the evoked fEPSP in hippocampal slices from adult mice exposed to nIH_MnTMPyP_ (Adult_nIH − Mn_) and from adult mice treated with MnTMPyP during recovery after neonatal IH exposure (Adult_REC − Mn_) prior to (1) and following (2) TBS. fEPSP slope plotted as a function of elapsed time and relative TBS in Adult_nIH − Mn_ and Adult_REC − Mn_ and the corresponding comparison 60 min after TBS (two-tailed *t*-test, *t* = 3.79; df = 8.16; *P* = 0.0051). **(E)** Representative immunoblot and corresponding quantification of GluN1 in hippocampal homogenates from Adult_control_, Adult_nIH_, Adult_nIH − Mn_, and Adult_REC − Mn_ [one-way ANOVA, *F* (3.16) = 0.13; *P* = 0.93; *N* = 5]. **(F)** Representative immunoblot and corresponding quantification of GluN2A in hippocampal homogenates from Adult_control_, Adult_nIH_, Adult_nIH − Mn_, and Adult_REC − Mn_ (one-way ANOVA, *F* (3.12) = 4.91; *P* = 0.0029, *N* = 4). **(G)** Representative immunoblot and corresponding quantification of GluN2B in hippocampal homogenates from Adult_control_, Adult_nIH_, Adult_nIH − Mn_, and Adult_REC − Mn_ [one-way ANOVA, *F* (3.12) = 4.91; *P* = 0.0019, *N* = 4]. **(H)** Comparison of the GluN2B to GluN2A ratio in hippocampal homogenates from Adult_control_, Adult_nIH_, Adult_nIH − Mn_, and Adult_REC − Mn_. The Bonferroni *post-hoc* tests were performed following one-way ANOVA. *P < 0.05, **P < 0.01, and N.S. = no significance, *P* ≥ 0.05. Scale bars: 10 ms x 0.2 mV.

To asses the consequences of nIH, we characterized LTP in adult mice that were allowed to recover from nIH for 6 weeks in room air (Adult_nIH_). In the hippocampal slices from Adult_nIH_, the magnitude of LTP was smaller compared to that in Adult_control_ preparations [Figure 5A; Adult_control_ (black): 167.58 ± 4.08% over baseline; *n* = 6 slices, *N* = 6 mice; Adult_nIH_ (red): 139.47 ± 2.76% over baseline; *n* = 7 slices, *N* = 7 mice; *P* = 0.003]. We next determined whether differences in LTP magnitude observed in Adult_nIH_ hippocampal slices were related to GluN2 subunit composition. In Adult_control_ slices, NMDAr-dependent LTP appeared to be largely dependent on the GluN2A containing NMDAr and independent of GluN2B-containing NMDAr as TCN-213 suppressed LTP (Figure 5B, orange, 119.53 ± 1.97% over baseline; *n* = 4 slices, *N* = 4 mice), while ifenprodil minimally affected LTP (Figure 5B, blue, 150.04 ± 3.59% over baseline; *n* = 4 slices, *N* = 4 mice; *P* = 0.0009). In contrast, LTP from Adult_nIH_ slices appeared to depend on GluN2B-containing NMDAr and be independent of GluN2A-containing NMDAr as ifenprodil blocked LTP (Figure 5C, blue: 108.1 ± 1.55 over baseline; *n* = 6, *N* = 5), while TCN-213 failed to block LTP (Figure 5C, orange, 132.08 ± 5.1 over baseline; *n* = 5, *N* = 3; *P* < 0.001).

As MnTMPyP prevented the immediate effects of nIH on LTP, we assessed how nIH_Mn_ and MnTMPyP administration following nIH (Adult_REC − Mn_) impacted LTP in adult hippocampal slices. In adult mice, the magnitude of LTP from the Adult_REC − Mn_ was smaller than the magnitude of LTP from Adult_nIH − Mn_ (Figure 5D, Adult_nIH − Mn_: green, 161.35 ± 8.39% over baseline; *n* = 6 slices, *N* = 6 mice; Adult_REC − Mn_: blue, 124.50 ± 4.87% over baseline; *n*= 7 slices, *N* = 7 mice; *P* = 0.0030).

We next examined biochemical and protein expression in the adult hippocampus. Neither HIF1a nor NOX isoforms were different from Adult_control_ in Adult_nIH_, Adult_nIH − Mn_, or Adult_REC − Mn_ (Supplementary Figure 1). Similarly, no differences in GluN1 subunit expression was found across experimental groups (Figure 5E, control = 1.00 ± 0.07; Adult_nIH_ = 0.99 ± 0.07; Adult_nIH − Mn_ = 1 ± 0.05 and Adult_REC − Mn_ = 0.95 ± 0.03, *N* = 5; *P* = 0.93). However, differences in both the subunit expression of GluN2A and GluN2B were evident. In Adult_nIH_ and Adult_REC − Mn_, GluN2A expression was suppressed when compared to Adult_control_; however, no difference was evident in Adult_nIH − Mn_ (Figure 5F, Adult_control_: 1 ± 0.05; Adult_nIH_: 0.56 ± 0.72; Adult_nIH − Mn_: 0.92 ± 0.07 and Adult_REC − Mn_: 0.71 ± 0.07, *N* = 4; *P* = 0.0029). While GluN2B expression was increased in Adult_nIH_, no differences were observed in Adult_nIH − Mn_ and Adult_REC − Mn_ (Figure 5G, Adult_control_: 1.02 ± 0.13; Adult_nIH_: 1.47 ± 0.11, Adult_nIH − Mn_: 1.14 ± 0.08, and Adult_REC − Mn_: 0.98 ± 0.13, *N* = 4; *P* =0.018). These changes coincided with a larger GluN2B:GluN2A ratio in adult mice exposed to nIH (Figure 5H, *P* = 0.0083).

### Spatial memory deficits occurring in adult mice previously exposed to nIH is mitigated by MnTMPyP

To determine whether the persistent changes in synaptic plasticity and NMDAr subunit expression due to neonatal IH corresponded with behavioral deficits related to spatial learning and memory, we next examined the performance of control adult mice (Adult_control_) and Adult_nIH_ and in the Barnes maze and Object Location task.

**Figure 6:**
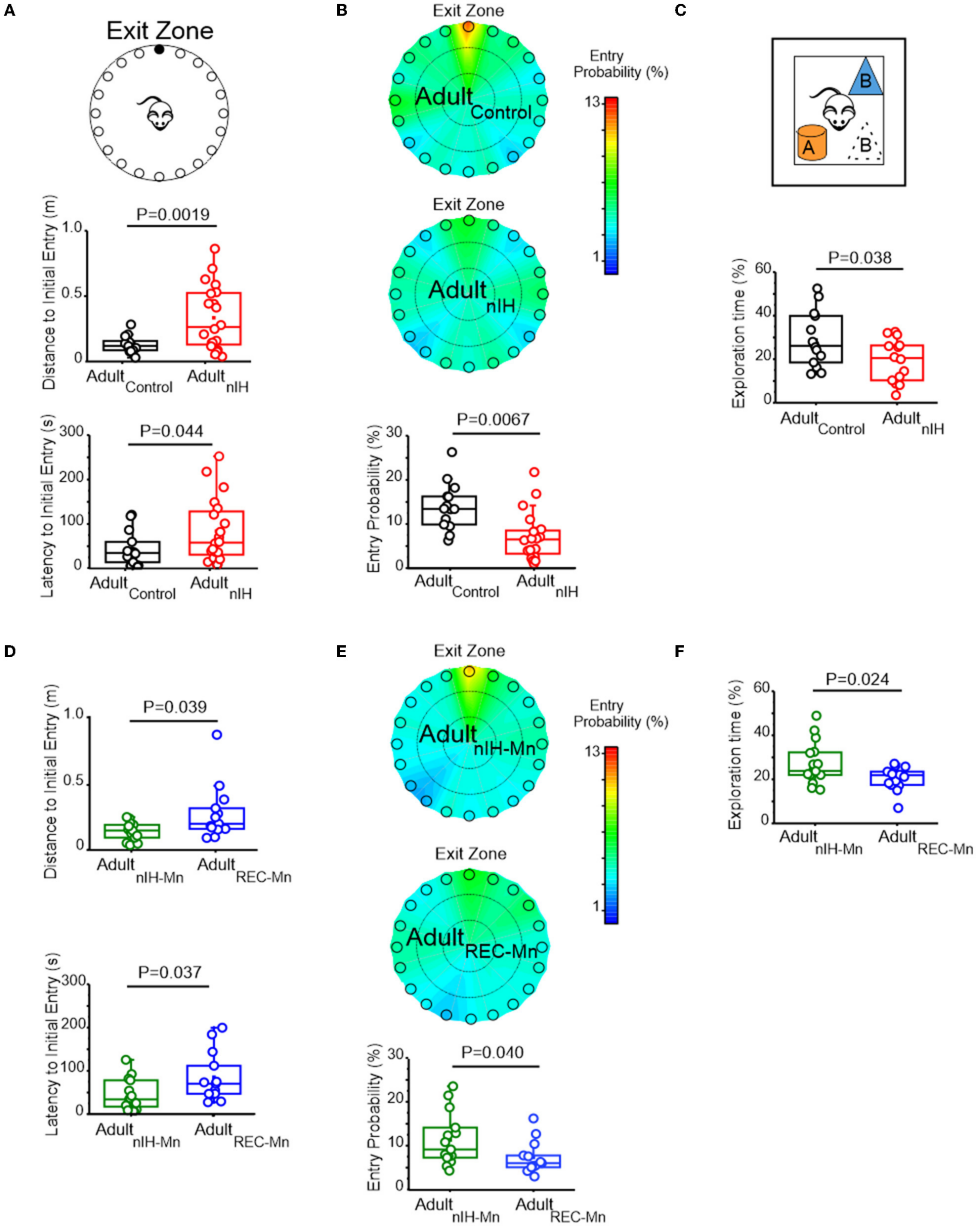
Adult mice exposed to nIH and received MnTMPyP after nIH exhibit spatial memory impairment. **(A)** The cartoon of the Barnes maze showing multiple false exits and a single exit (black circle) that mice are trained to enter and exit the maze. Comparisons of distance to initial entry (*t* = 3.51, df = 22.57; *P* = 0.0019) and latency to initial entry (*t* = 2.10, df = 30.54; *P* = 0.044) into the exit zone during the PROBE trial of the Barnes maze. Performance during training sessions was similar for all experimental groups. See Table 2 and Supplementary material 2 for additional details. **(B)** Heat maps of mean entry probability in Adult_control_ and Adult_nIH_ and the corresponding comparison of entry probability into the exit zone during the PROBE trial of the Barnes maze (*t* = 2.92, df = 28.45; *P* = 0.0067). **(C)** Cartoon of the Object Location task during the PROBE trial. Comparisons of exploration time during the PROBE trial of the Object Location task. (*t* = 2.18, df = 24.53; *P* = 0.038). Performance during the familiarization session was similar for all experimental groups. See Table 3 and Supplementary material 3 for additional details. **(D)** Comparison of distance to initial entry in Adult_REC − Mn_ and Adult_nIH − Mn_ (*t* = 2.27, df = 14.00; *P* = 0.039) and comparison of latency to initial entry Adult_nIH − Mn_ vs. Adult_REC − Mn_ (*t* = 2.23, df = 19.37; *P* = 0.037) during the PROBE trial of the Barnes Maze. Performance during training sessions was similar for all experimental groups. See Table 4 and Supplementary material 2 for additional details. **(E)** Heat maps of the mean entry probability in Adult_nIH − Mn_ and Adult_REC − Mn_ and the corresponding comparison of entry probability into the exit zone during the PROBE trial of the Barnes Maze (*t* = 2.17, df = 23.91; *P* = 0.040). **(F)** Comparison of exploration time between Adult_Mn_ and Adult_REC − Mn_ during the PROBE trial of the object location test (*t* = 2.52, df = 14; *P* = 0.024). Performance during the familiarization session was similar for all experimental groups. See Table 5 and Supplementary material 3 for additional details.

In the Barnes maze test, both velocity and distance traveled during the training sessions were similar in Adult_control_ (*N* = 14) and Adult_nIH_ (*N* = 20) (Table 2). Additionally, both Adult_control_ and Adult_nIH_, exhibited a progressive decrease in total latency to exit the maze across the training sessions (Supplementary Figure 2). During the PROBE trial, the initial distance (Figure 6A; control: 0.13 ± 0.017 m vs. Adult_nIH_: 0.33 ± 0.05 m, *P* = 0.0019) and latency to initial entry (Figure 6A; control: 43.04 ± 10.44 s vs. Adult_nIH_: 83.13 ± 15.99 s, *P* = 0.04) into the exit zone were greater in Adult_nIH_. These differences were accompanied by a smaller entry probability into the exit zone in Adult_nIH_ (Figure 6B; control: 12.63 ± 1.42% vs. Adult_nIH_: 7.14 ± 1.21%, *P* = 0.0067).

**Table 2:** Velocity and distance traveled during the Barnes maze training sessions.

	**Adult_control_ (*N* = 14)**	***P*-value**	**Adult_nIH_/Adult_nIH − Saline_ (*N* = 20)**	***P*-value**
Velocity (m/s)	Session 1: 0.058 ± 0.003 Session 2: 0.053 ± 0.005 Session 3: 0.049 ± 0.005	*P* = 0.39	Session 1: 0.06 ± 0.006 Session 2: 0.054 ± 0.005 Session 3: 0.055 ± 0.005	*P* = 0.69
Distance traveled (m)	Session 1: 18.53 ± 2.05 Session 2: 8.68 ± 1.20 Session 3: 4.8 ± 1.48	*P* < 0 .001	Session 1: 20.58 ± 2.56 Session 2: 12.27 ± 1.53 Session 3: 7.36 ± 1.73	*P* = 0.0006

During the open field session in the object location task, Adult_control_ (*N* = 14) and Adult_nIH_ (*N* = 14) exhibited similar velocities, distance traveled, time in the periphery, and time in the center (Table 3), suggesting no locomotor differences between groups. During the familiarization session, control and Adult_nIH_ both exhibited a progressive decrease in exploration times and similar exploration times with both objects (Supplementary Figure 3). These data suggested that both groups learned the location of both objects and did not have a preference for either of the objects tested. During the PROBE trial of the Object Location task, control mice exhibited greater exploration time compared to nIH (Figure 6C: control = 28.81 ± 3.38% and nIH = 19.42 ± 2.63%; *P* = 0.038).

**Table 3:** Open field locomotor activity in Adult_control_ and Adult_nIH_/Adult_nIH − Saline_.

	**Adult_control_ (*N* = 14)**	**Adult_nIH_/Adult_nIH − Saline_ (*N* = 14)**	***P*-value**
Velocity (m/s)	0.05 ± 0.003	0.06 ± 0.004	*P =* 0.30
Distance traveled (m)	33.16 ± 2	32.81 ± 1.65	*P =* 0.89
Time in the periphery (s)	361.3 ± 21.26	323.5 ± 18.16	*P =* 0.18
Time in the center (s)	237.8 ± 21.22	285.6 ± 23.13	*P =* 0.12

We sought to determine whether the adult behavioral performance was impacted by MnTMPyP administration during nIH (i.e., Adult_nIH − Mn_) or by MnTMPyP administration following nIH (i.e., Adult_REC − Mn_). Both Adult_nIH − Mn_ (*N* = 16) and Adult_REC − Mn_ (*N* = 13) exhibited progressive reductions in the distance traveled across training sessions without changed velocities (Table 3). These reductions corresponded with progressive improvement in exiting the Barnes maze during the three training sessions (Table 2). During the PROBE trial, the initial distance (Figure 6D; Adult_nIH − Mn_: 0.13 ± 0.016 m vs. Adult_REC − Mn_: 0.27 ± 0.05 m, *P* = 0.039) and latency to initial entry (Figure 6D; Adult_nIH − Mn_: 43.83 ± 9.03 s vs. Adult_REC − Mn_: 84.56 ± 15.79s, *P* = 0.037) into the exit zone were lower in Adult_nIH − Mn_ when compared to Adult_REC − Mn_. Adult_nIH − Mn_ also showed a greater entry probability into the exit zone when compared to Adult_REC − Mn_ (Figure 6E; Adult_nIH − Mn_: 11.45 ± 1.52% vs. Adult_REC − Mn_: 7.43 ± 5.18%, *P* = 0.040).

During the open field session of the Object Location task, Adult_nIH − Mn_ (*N* = 16) and Adult_REC − Mn_ (*N* = 13) exhibited similar velocities, distance traveled, time in the periphery, and time in the center (Table 3), suggesting no locomotor differences between groups. During the familiarization session, Adult_nIH − Mn_ and Adult_REC − Mn_ both also exhibited a progressive decrease in exploration times (Table 4) and similar times exploring both objects (Table 5). However, during the PROBE trial of the Object Location task, exploration time for the moved object (i.e., object B) was greater in Adult_nIH − Mn_ when compared to Adult_REC − Mn_ (Figure 6F; Adult_nIH − Mn_: 27.21 ± 2.51 % vs. Adult_REC − Mn_: 20.44 ± 1.31 %, *P* = 0.024)

**Table 4:** Velocity and distance of MnTMPyP-treated mice during the Barnes maze training sessions.

	**Adult_nIH − Mn_ (*N* = 16)**	***P*-value**	**Adult_REC − Mn_ (*N* = 13)**	***P*-value**
Velocity (m/s)	Session 1: 0.07 ± 0.001 Session 2: 0.07 ± 0.003 Session 3: 0.064 ± 0.008	*P =* 0.76	Session 1: 0.07 ± 0.02 Session 2: 0.07 ± 0.004 Session 3: 0.065 ± 0.012	*P =* 0.87
Distance traveled (m)	Session 1: 18.29 ± 1.05 Session 2: 10.42 ± 1.28 Session 3: 5.32 ± 0.82	*P* < 0.0001	Session 1: 15.32 ± 2.68 Session 2: 7.84 ± 1.31 Session 3: 8.25 ± 2.14	*P =* 0.043

**Table 5:** Open field locomotor activity in MnTMPyP-treated subjects.

	**Adult_nIH − Mn_ (*N* = 16)**	**Adult_REC − Mn_ (*N* = 13)**	***P*-value**
Velocity (m/s)	0.05 ± 0.003	0.055 ± 0.002	*P =* 0.43
Distance traveled (m)	35.21 ± 1.45	33.25 ± 1.24	*P =* 0.33
Time in the periphery (s)	316.7 ± 26.31	304.1 ± 27.19	*P =* 0.95
Time in the center (s)	272 ± 24.65	280.2 ± 27.12	*P =* 0.83

## Discussion

Intermittent hypoxia can be experienced across a lifetime. While commonly associated with untreated sleep apnea in adolescents (Narang and Mathew, 2012) and adults (Ramirez et al., 2013), intermittent hypoxia may also be experienced by children suffering from autonomic dysautonomias, such as that observed in Rett Syndrome and other conditions (Glaze et al., 1987; Carroll et al., 2015; Ramirez et al., 2020). In perinatal life, intermittent hypoxia may be experienced embryonically when maternal sleep apnea is left untreated (Dominguez et al., 2018). Whereas, in premature infants, it occurs with apneas of prematurity (Martin et al., 2011). Each of these conditions have associations with neurocognitive impairment (Ramirez et al., 2013; Poets, 2020; Slattery et al., 2023), leading to the hypothesis that intermittent hypoxia (IH) exposure, independent of life stage, negatively affects neurophysiology and behavior. Over the past three decades, the majority of research examining this hypothesis has been almost exclusively focused on examining how IH affects neurophysiology in the adult brain. Consistent with the proposal that unstable breathing associated with premature birth promotes oxidative stress to cause disturbances in neonatal brain, our study presents a role for the nIH-dependent prooxidant state to remodel NMDAr subunit composition and to cause deficits in synaptic plasticity in the hippocampus. These phenomena are evident immediately following nIH and persist later in life where they coincide with behavioral deficits. We demonstrated that antioxidant administration has a discrete window to be effective in preventing the nIH-dependent phenomena on NMDAr subunit expression, hippocampal synaptic plasticity, and behavioral changes. As subjects with no known genetic predisposition for neurophysiological or behavioral deficits were used, our findings highlight the critical nature of stable oxygen homeostasis in normal brain development and support the view that neonatal intermittent hypoxia is an independent risk factor for negatively impacting neurophysiological development and behavior later in life.

Recent advancements in understanding how IH affects synaptic plasticity and neurogenesis in the adult hippocampus have established a mechanistic framework by which IH-dependent HIF1a signaling promotes a prooxidant condition (Chou et al., 2013; Arias-Cavieres et al., 2020; Khuu et al., 2021). Such an IH-dependent prooxidant condition suppresses neurogenesis (Khuu et al., 2019, 2021) and impairs synaptic plasticity (Khuu et al., 2019; Arias-Cavieres et al., 2020, 2021). Similar to our findings with nIH, the IH-dependent phenomena occurring in the adult hippocampus are mitigated with the administration of MnTMPyP treatment during IH exposure (Khuu et al., 2019; Arias-Cavieres et al., 2020, 2021). MnTMPyP acts as a superoxide anion scavenger (Faulkner et al., 1994) suggesting that excess superoxide anion and/or other related reactive species, such as hydrogen peroxide, play a central role initiating effects of nIH in the hippocampus. This is further supported by the observation that the nIH-related prooxidant state coincided with enhanced NOX isoform expression. Both NOX2 and NOX4 produce reactive oxygen species, and the expression of both has been associated with IH-dependent HIF1a activity (Peng et al., 2006, 2014; Chou et al., 2013; Arias-Cavieres et al., 2020). In addition to promoting ROS production, nIH may also suppress antioxidant defenses. Indeed, nIH causes the epigenetic suppression of superoxide anion 2 elsewhere in the nervous system (Nanduri et al., 2012). However, the extent to which the depletion of hippocampal antioxidant defenses occurs with nIH and contributes to hippocampal remodeling remains to be further resolved.

In the adult hippocampus, IH downregulates the obligatory subunit of the NMDAr, as observed in the adult hippocampus (Arias-Cavieres et al., 2020, 2021). However, in the neonatal hippocampus, the IH-dependent prooxidant state perturbs the normal developmental transition of expression predominance from the GluN2B subunit to the GluN2A subunit. The conversion of GluN2 subunit predominance from GluN2B to GluN2A in the brain normally occurs during early postnatal development. In the primary visual cortex of dark-reared animals, GluN2 subunit remodeling is rapidly precipitated by a single 1-h exposure to a visual stimulus (Quinlan et al., 1999; Philpot et al., 2001), demonstrating that GluN2 subunit dominance can be shaped by early life experience. Consistent with this view, experiencing short repeated oscillations in oxygenation in early postnatal effectively influences the transition in GluN2 subunit identity. Additionally, our findings showed that this experience-dependent phenomenon occurring in early life persists to affect hippocampal synaptic properties and associated behavioral outcomes later in life.

GluN2 subunit composition dictates the biophysical, pharmacological, and signaling properties of the NMDAr (Cull-Candy and Leszkiewicz, 2004; Traynelis et al., 2010; Paoletti et al., 2013; Vyklicky et al., 2014), which, in turn, influences synaptic timing and summation properties (Lei and Mcbain, 2002; Kumar and Huguenard, 2003; Paoletti et al., 2013). Thus, nIH-dependent changes to GluN2 subunit composition would be predicted to affect the NMDAr-dependent physiology. Indeed, the nIH-dependent deficits to LTP appear to be related to changed GluN2 subunit composition as LTP following nIH was prevented by blocking the GluN2B-containing NMDAr but was insensitive to the blockade of the GluN2A containing NMDAr. As CAMKII activity is associated with GluN2B-containing NMDAr (Paoletti et al., 2013; Tang et al., 2020; Hosokawa et al., 2021), the continued predominance of GluN2B activity may increase CAMKII activity and subsequently affect hippocampal neurophysiology and associated behaviors. MnTMPyP treatment during nIH effectively mitigated the nIH-driven downregulation of GluN2A receptors but also prevented the effects on LTP and behavioral performance. Although further resolution is required to determine how GluN2B gain of function and GluN2A loss of function each contribute to the persistence of neurophysiological deficits caused by nIH, our findings demonstrated that oxidative stress and GluN2A subunit downregulation are significant factors contributing to immediate and long-term deficits observed in synaptic plasticity and behavior following nIH.

While treating with MnTMPyP mitigated the prooxidant state and prevented both immediate and long-term consequences of nIH, MnTMPyP administration following nIH neither prevented GluN2A downregulation nor impaired synaptic plasticity. When compared to adult mice that received MnTMPyP during nIH, adult mice treated with MnTMPyP following nIH also exhibited memory deficits. Additionally, neither nuclear HIF1a nor NOX isoforms are elevated in adult hippocampal tissue following nIH. These findings strongly suggest that, while the prooxidant state and enhanced HIF1a signaling may initially drive the immediate nIH-dependent changes in the neonatal hippocampus, the persistent effects of nIH on the hippocampus and behavior later in life do not appear driven by a persistent elevation in HIF1a signaling or a prooxidant state, given that no differences were observed in adult mice in HIF1a, NOX isoforms, or MDA content. Rather, the pro-oxidant activity may initiate epigenetic changes to cause lasting effects on hippocampal NMDAr subunit identity, synaptic plasticity, and behavioral changes later in life. Interestingly, offspring exposed to gestational IH have improved AMPA receptor activity in the prefrontal cortex (Vanderplow et al., 2022). This remodeling appears to involve dysregulation in postnatal signaling related to the mammalian target of rapamycin (mTOR) pathway (Vanderplow et al., 2022). It is possible that dysregulated mTOR signaling may also be involved with the nIH-mediated remodeling of the hippocampal GluN2 subunit composition. Thus, while we provide key insights into the impact of nIH on NMDAr-related processes and the underlying factors that drive such changes, a need exists to further resolve the potential consequences of nIH on other aspects of hippocampal synaptic physiology but also provide a deeper understanding of the molecular signaling by which nIH remodels hippocampal properties.

In conclusion, understanding the mechanistic role that intermittent hypoxia has on neurodevelopmental outcomes remains limited when compared to the depth of knowledge into the genetic and molecular determinants of intellectual disability associated with neurodevelopmental disorders. Advancements toward understanding and addressing the consequences of mutations such as SYNGAP1 (Hamdan et al., 2009; Clement et al., 2012; Zoghbi and Bear, 2012; Yang et al., 2023), Fragile X Messenger Ribonucleoprotein 1 (Leal et al., 2014; D'incal et al., 2022; Monday et al., 2022), and MECP2 (Banerjee et al., 2019) continue to grow, yet the severity of neurodevelopmental impairment often varies among individuals with such genetic predispositions. We have identified a mechanism by which intermittent hypoxia linked unstable breathing during early postnatal life may independently lead to negative long-term neurocognitive outcomes. Such a mechanism may augment intellectual deficits among individuals genetically or otherwise predisposed to neurodevelopmental impairment.

## Data availability statement

The raw data supporting the conclusions of this article will be made available by the authors, without undue reservation.

## Ethics statement

The animal study was reviewed and approved by Institutional of Animal Care and Use Committee at the University of Chicago.

## Author contributions

AA-C and AG conceived, designed experiments, wrote, and revised the manuscript. AA-C performed experiments and conducted analyses. AG provided reagents and support. All authors contributed to the article and approved the submitted version.

## Supplementary material

Supplementary Figure 1 Adult mice exposed to IH do not have increased HIF1a, NOX2 and NOX4 expression. **(A)** (*left*) Representative immunoblot and corresponding comparison of nuclear HIF1a Adult_control_, Adult_nIH_, Adult_nIH − Mn_, and Adult_REC − Mn_ [one-way ANOVA, *F* (3.20) = 1.14; *P* = 0.35, *N* = 6]. **(B)** Representative immunoblot and corresponding comparison of nuclear NOX2 Adult_control_, Adult_nIH_, Adult_nIH − Mn_, and Adult_REC − Mn_ [one way ANOVA, *F* (3.12) = 0.35; *P* = 0.78, *N* = 4]. **(C)** Representative immunoblot and corresponding comparison of nuclear NOX4 Adult_control_, Adult_nIH_, Adult_nIH − Mn_, and Adult_REC − Mn_ [one way ANOVA, *F* (3.12) = 0.79; *P* = 0.51, *N* = 4]_._ A Bonferroni *post-hoc* test was performed following one-way ANOVA. N.S = no significance, *P* ≥ 0.05.

Supplementary Figure 2 Behavioral performance during training in the Barnes maze test is evident in all experimental groups. **(A)** During the training sessions, the mean total latency to exit the maze progressively decreased in Adult_control_ [*F* (2.39) = 18.47, *P* < 0.0001, *N* = 14] and Adult_nIH_ [*F* (2.57) = 10.98, *P* = 0.0015, *N* = 20]. The black line represents the mean latency per trial, whereas the gray and red lines represent individual performance during training. **(B)** The black line represents mean latency per trial, whereas the green and blues lines represent individual latency when training Adult_nIH − Mn_ [*F* (2.45) = 31.55, *P* < 0.0001, *N* = 16] and Adult_REC − Mn_ [*F* (2.36) = 9.14, *P* = 0.0006, *N* = 13]. The values indicate mean ± S.E. A one-way ANOVA was performed for A and B.

Supplementary Figure 3 Exploration of objects during familiarization in the object location test is similar in all experimental groups. **(A)** Object Location diagram. **(B)** The exploration time of object for both objects progressively decreased in Adult_control_ [*F* (2.39) = 39.05, *P* < 0.001; *N* = 14] and Adult_nIH_ [*F* (2.39) = 18.33), *P* < 0.0001; *N* = 14] in the training sessions during familiarization. The black line represents the mean exploration time during the training sessions, whereas the gray and red represent individual performance during training. **(C)** (*left*) Adult_Control_ have similar exploration times with each object in the first (*t* = 0.80, df = 23.94; *P* = 0.47), second (*t* = 1.52, df = 22,63; *P* = 0.23), and third (*t* =1.36, df = 24; *P* = 0.22) sessions. (*right*) Adult_nIH_ have similar exploration times with each object in the first session (*t* = 0.06, df = 17.08; *P* = 0.99), second session (*t* = 1.34, df = 17.79; *P* = 0.11), and third sessions (*t* = 0.06, df = 15.92; *P* = 0.44). **(D)** The exploration time for both objects decreased in Adult_nIH − Mn_ [*F* (2.39) = 6.10, *P* = 0.0049, *N* = 16] and Adult_REC − Mn_ [*F* (2.33) = 14.33, *P* < 0.0001, *N* = 13]. The black line represents the mean exploration time during the training sessions, whereas the green and blue lines represent individual performance during training. **(E)** (*left*) Adult_nIH − Mn_ have similar exploration times with each object in the first (*t* = 0.74, df = 29.62; *P* = 0.46), second (*t* = 0.008, df = 29.09; *P* = 0.99), and third (*t* = 0.78, df = 26.35; *P* = 0.43) sessions. (*right*) Adult_REC − Mn_ have similar exploration times with each object in the first (*t* = 0.28, df = 26.09; *P* = 0.77), second (*t* = 0.62, df = 28.00; *P* = 0.53), and third (*t* = 1.88, df = 20.67; *P* = 0.07) sessions. A one-way ANOVA was performed for **(B, D)**. A paired two-sided test was performed for **(C, E)**.
