# Supplementary figures and images for: A consequence of immature breathing induces persistent changes in hippocampal synaptic plasticity and behavior: a role of prooxidant state and NMDA receptor imbalance
Source: Front Mol Neurosci. 2023 Jun 29;16:1192833. doi: 10.3389/fnmol.2023.1192833 (PMC10338931; doi:10.3389/fnmol.2023.1192833)

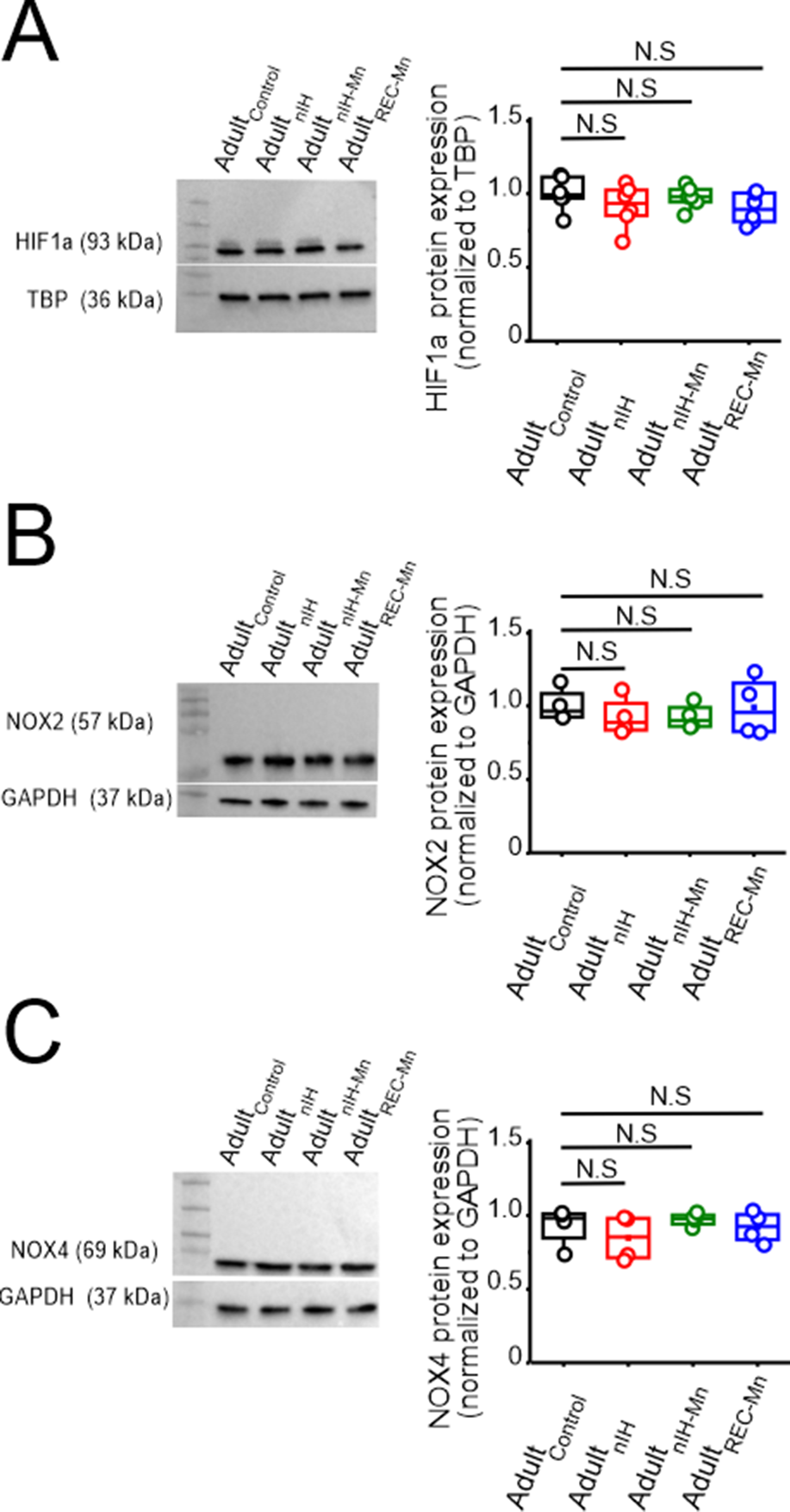

Supplement: Supplementary Figure 1 — Adult mice exposed to IH do not have increased HIF1a, NOX2 and NOX4 expression. (A) (left) Representative immunoblot and corresponding comparison of nuclear HIF1a Adultcontrol, AdultnIH, AdultnIH − Mn, and AdultREC − Mn [one-way ANOVA, F (3.20) = 1.14; P = 0.35, N = 6]. (B) Representative immunoblot and corresponding comparison of nuclear NOX2 Adultcontrol, AdultnIH, AdultnIH − Mn, and AdultREC − Mn [one way ANOVA, F (3.12) = 0.35; P = 0.78, N = 4]. (C) Representative immunoblot and corresponding comparison of nuclear NOX4 Adultcontrol, AdultnIH, AdultnIH − Mn, and AdultREC − Mn [one way ANOVA, F (3.12) = 0.79; P = 0.51, N = 4]. A Bonferroni post-hoc test was performed following one-way ANOVA. N.S = no significance, P ≥ 0.05. [file Image_1.TIF]

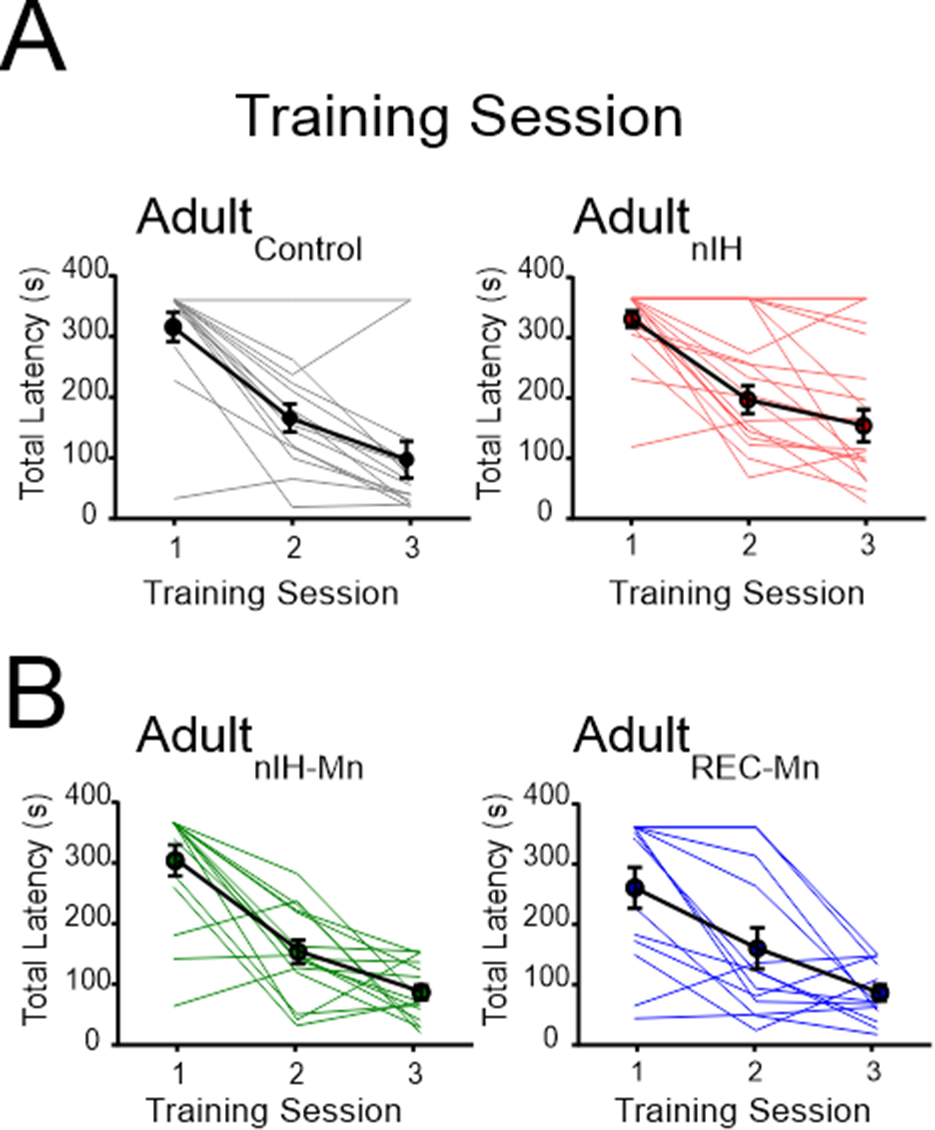

Supplement: Supplementary Figure 2 — Behavioral performance during training in the Barnes maze test is evident in all experimental groups. (A) During the training sessions, the mean total latency to exit the maze progressively decreased in Adultcontrol [F (2.39) = 18.47, P < 0.0001, N = 14] and AdultnIH [F (2.57) = 10.98, P = 0.0015, N = 20]. The black line represents the mean latency per trial, whereas the gray and red lines represent individual performance during training. (B) The black line represents mean latency per trial, whereas the green and blues lines represent individual latency when training AdultnIH − Mn [F (2.45) = 31.55, P < 0.0001, N = 16] and AdultREC − Mn [F (2.36) = 9.14, P = 0.0006, N = 13]. The values indicate mean ± S.E. A one-way ANOVA was performed for A and B. [file Image_2.TIF]

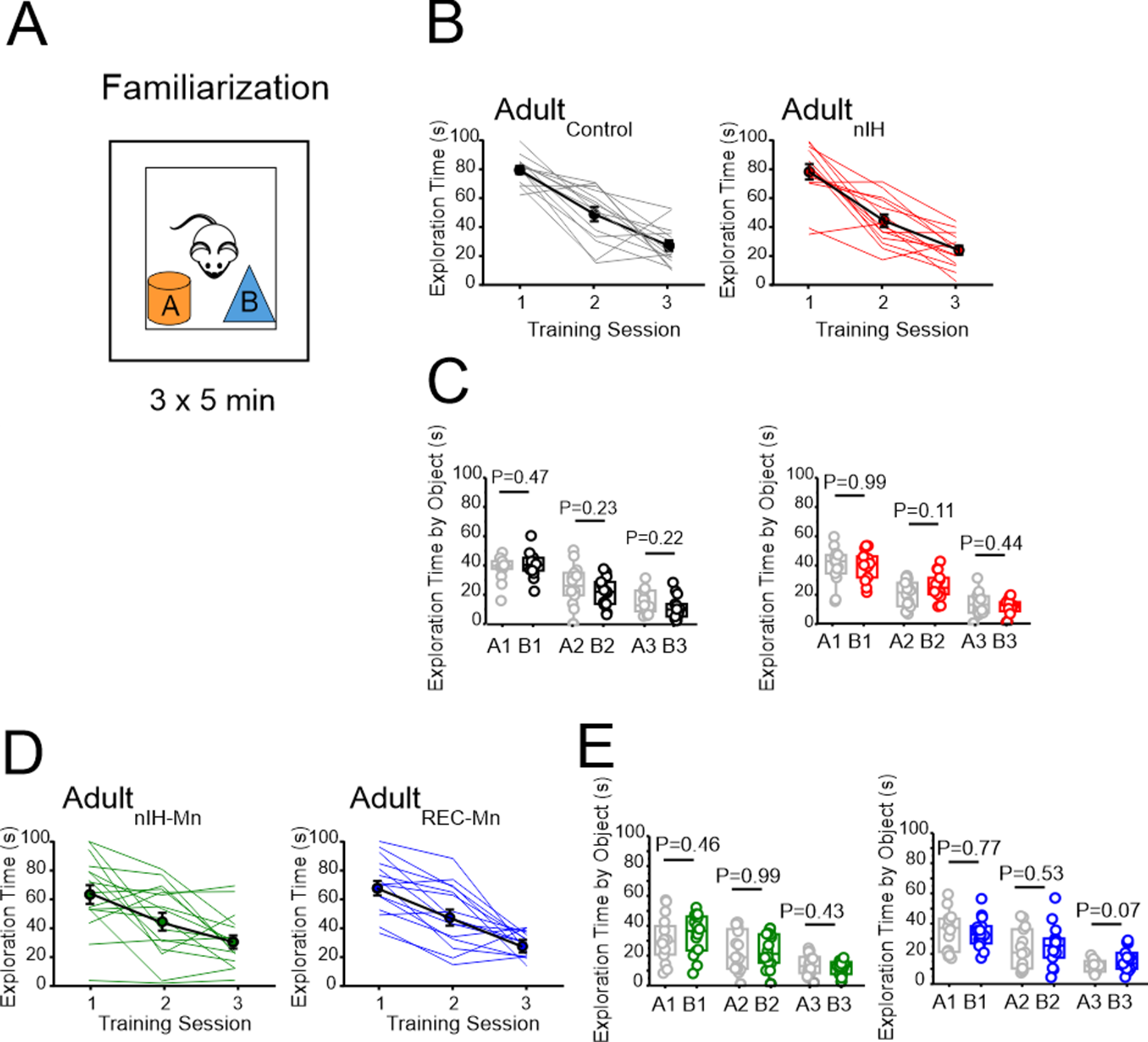

Supplement: Supplementary Figure 3 — Exploration of objects during familiarization in the object location test is similar in all experimental groups. (A) Object Location diagram. (B) The exploration time of object for both objects progressively decreased in Adultcontrol [F (2.39) = 39.05, P < 0.001; N = 14] and AdultnIH [F (2.39) = 18.33), P < 0.0001; N = 14] in the training sessions during familiarization. The black line represents the mean exploration time during the training sessions, whereas the gray and red represent individual performance during training. (C) (left) AdultControl have similar exploration times with each object in the first (t = 0.80, df = 23.94; P = 0.47), second (t = 1.52, df = 22,63; P = 0.23), and third (t =1.36, df = 24; P = 0.22) sessions. (right) AdultnIH have similar exploration times with each object in the first session (t = 0.06, df = 17.08; P = 0.99), second session (t = 1.34, df = 17.79; P = 0.11), and third sessions (t = 0.06, df = 15.92; P = 0.44). (D) The exploration time for both objects decreased in AdultnIH − Mn [F (2.39) = 6.10, P = 0.0049, N = 16] and AdultREC − Mn [F (2.33) = 14.33, P < 0.0001, N = 13]. The black line represents the mean exploration time during the training sessions, whereas the green and blue lines represent individual performance during training. (E) (left) AdultnIH − Mn have similar exploration times with each object in the first (t = 0.74, df = 29.62; P = 0.46), second (t = 0.008, df = 29.09; P = 0.99), and third (t = 0.78, df = 26.35; P = 0.43) sessions. (right) AdultREC − Mn have similar exploration times with each object in the first (t = 0.28, df = 26.09; P = 0.77), second (t = 0.62, df = 28.00; P = 0.53), and third (t = 1.88, df = 20.67; P = 0.07) sessions. A one-way ANOVA was performed for (B, D). A paired two-sided test was performed for (C, E). [file Image_3.TIF]
